# To B or Not to B: Understanding B Cell Responses in the Development of Malaria Infection

**DOI:** 10.3389/fimmu.2018.02961

**Published:** 2018-12-14

**Authors:** Eduardo L. V. Silveira, Mariana R. Dominguez, Irene S. Soares

**Affiliations:** Department of Clinical and Toxicological Analyses, School of Pharmaceutical Sciences, University of São Paulo, São Paulo, Brazil

**Keywords:** B cell biology, malaria, antibodies, effective mechanism, protective immunity

## Abstract

Malaria is a widespread disease caused mainly by the *Plasmodium falciparum* (Pf) and *Plasmodium vivax* (Pv) protozoan parasites. Depending on the parasite responsible for the infection, high morbidity and mortality can be triggered. To escape the host immune responses, *Plasmodium* parasites disturb the functionality of B cell subsets among other cell types. However, some antibodies elicited during a malaria infection have the potential to block pathogen invasion and dissemination into the host. Thus, the question remains, why is protection not developed and maintained after the primary parasite exposure? In this review, we discuss different aspects of B cell responses against *Plasmodium* antigens during malaria infection. Since most studies have focused on the quantification of serum antibody titers, those B cell responses have not been fully characterized. However, to secrete antibodies, a complex cellular response is set up, including not only the activation and differentiation of B cells into antibody-secreting cells, but also the participation of other cell subsets in the germinal center reactions. Therefore, a better understanding of how B cell subsets are stimulated during malaria infection will provide essential insights toward the design of potent interventions.

## Malaria Infection and Immunity

Malaria is a widespread disease mainly caused by the *Plasmodium falciparum* (Pf) and *Plasmodium vivax* (Pv) parasites in tropical countries. Currently, half of the world population lives in areas at risk of a malaria infection. In 2016, a global estimative enumerated 216 million clinical cases and 445,000 deaths associated with this disease (1), portraying the real magnitude of this public health problem. Most cases of malaria morbidity and mortality have been attributed to Pf infections, prevalent in sub-Saharan Africa and characterized by high parasitemias and severe complications, especially in children (2). Contrarily, Pv infections are more disseminated in American and Asian countries and induce lower parasitemia levels and milder symptoms. Rarely, Pv infections can elicit severe symptoms and kill like Pf infections (2–4).

*Plasmodium* parasites have a complex life cycle, with sporozoites transmitted from the *Anopheles* mosquito salivary glands to the human skin dermis during mosquito blood meals. These motile parasites cross layers of the skin and enter the bloodstream, reaching the liver within hours upon infection. Then, they invade the hepatocytes, replicating and differentiating into schizonts. In the case of a Pv infection, part of the sporozoites are transformed into dormant forms called hypnozoites, which can be activated even after a long term of parasite infection. As a result of the hepatocyte burst, the merozoites are released in the bloodstream and invade the erythrocytes (Pf parasites) or the reticulocytes (Pv parasites), initiating the asexual blood stage of the cycle. These parasitic forms undergo several rounds of multiplication and differentiation, increasing the parasitemia levels in the host. Those forms found in infected red blood cells (iRBCs) have been identified as rings, trophozoites, schizonts, and gametocytes. Whereas the newly-released merozoites can keep re-invading the erythrocytes, a small fraction of them differentiate directly into gametocytes, giving rise to the sexual blood stage. Gametocytes are ingested during the mosquito blood meal and fuse to each other within the digestive tract, forming a zygote. The zygote differentiates into an ookinete, followed by oocyst forms, previously to the generation of infectious sporozoites that can be found in a mosquito's salivary glands (5, 6). Interestingly, the bone marrow has been described as the major parasite reservoir for early blood stage (asexual and sexual) and gametocytes in Pv infections (7, 8).

Regarding the mechanisms of immunity naturally induced by malaria, the humoral response has been described as the most important for the establishment of protection. This concept has been solidified after the finding that a passive transfer of serum samples from malaria-immune adults controlled the Pf parasitemia levels and ameliorated symptoms in acutely infected children (9). Although the elicitation of the humoral response is critical to reduce malaria morbidity and mortality, antibody-dependent protective immunity usually takes multiple parasitic exposures and may take even years to be established. The extensive genetic diversity of clinical Pf and Pv malaria episodes (10, 11) and the low frequency of malaria-specific memory B cells (MBCs) detected in residents of high endemic areas (12, 13) corroborate this statement. Considering that antibodies represent a snapshot of B cell responses at a single cell level (14), it is fundamental to understand how this cellular component is stimulated upon *Plasmodium* infection to improve vaccine formulations and consequently generate more effective antibodies against human malaria. In this review, we present the distinct aspects of B cell immunity derived from a malaria infection, ranging from the activation of naive B cells to the generation of antibody-secreting cells and the mechanisms of action by protective antibodies.

## Malaria-specific B cell Responses

During malaria infection, thousands of parasitic antigens are expressed in each stage of the parasite life cycle (15). However, the anti-malarial humoral responses are preferentially headed to blood stage antigens rather than the liver counterparts. Besides the differences on the antigen density, a malaria murine model has shown that the blood stage of infection weakens the humoral immunity against the liver stage antigens through the modification of lymphoid structures and the expression of cytokines and chemokines (16). Overall, these responses are mainly characterized by the generation of the antibody-secreting cells (ASCs), memory B cells (MBCs), and antibody titers. Whereas, the MBCs and antibody titers have been found with steady levels for years in individuals living in low Pf and Pv malaria-endemic areas without the evidence of reinfection (17), these parameters are not sustained for longer periods, especially in younger individuals from high Pf-endemic areas (12, 13).

The question arises, disregarding the timing that antibodies can be detected in serum samples, how is their secretion triggered upon malaria infection? Usually, the antigen-specific antibodies are expected to be detected in the serum in < 2 weeks upon any pathogen exposure. During this period, the naive B cells are activated upon B cell receptor (BCR) interaction with a parasitic antigen in the periphery, eliciting cell proliferation and differentiation into multiple subsets such as the MBCs, follicular B cells (FoBs), major players of the germinal center (GC) reactions, or marginal zone B cells (MZBs). Although all these B cell subsets express immunoglobulin (Ig) genes, only the ASCs secrete antibodies. Regarding FoBs, they form and maintain structures called the germinal centers (GCs) together with the follicular T helper cells (TFh), dendritic cells (FDCs), cytokines [IL-21, IL-6, and B cell activating factor (BAFF)], and the critical participation of co-stimulatory molecules (CD40L and ICOS). During the germinal center reactions, the GC B cells are activated and undergo several rounds of antigen selection, acquiring a mature status through the somatic hypermutations and class-switch in Ig genes. Thus, it prompts the production of high-affinity, class-switched antibodies. Models of human and murine malaria infections point to higher numbers of GC B cells and lower for MZB-like cells (18, 19). Terminal signaling triggered by activation guides the FoB cells to exit the follicles and differentiate into high-affinity, atypical, or classical MBCs (20) or short-lived, class-switched ASCs. However, there is evidence from a malaria murine model that high affinity, somatic hypermutated IgM+ MBCs dominate a recall response, being differentiated either into IgM+ or IgG+ ASCs and MBCs (21). The higher the frequency of the antigen-specific MBCs during that second encounter with the antigen, the higher will be the frequency of the antigen-specific ASCs generated. It is still controversial whether the unswitched or switched MBCs enter the GCs or form the ASCs: [reviewed by (22)]. Once generated, the ASCs migrate through circulation to the bone marrow or secondary lymphoid organs. More specifically, their physical contact with bone marrow stroma cells and the recognition of cytokines described above lead to modifications in their transcriptome profile, upregulating preferentially the expression of anti-apoptotic genes. This process culminates in their transformation from short-lived to long-lived ASCs, whose function results in the increased titers of serum antibodies (23).

On the other hand, malaria infection affects the generation of some critical cell subsets for humoral responses. Repeated parasitic exposures drive the expansion and accumulation of atypical MBCs in individuals from Pf malaria-endemic areas (24, 25). Although these cells have been mostly associated with the impaired B cell responses in this infection context, some groups have stated that atypical MBCs present a similar function as classical MBCs (26). Furthermore, this was demonstrated in an impaired GC response in a murine model of severe malaria due to the inhibition of TFh cell differentiation (27). In addition, the murine conventional DCs presented lower BAFF expression, culminating in a reduced ASC number in the spleen (28). Noteworthily, the bone marrow ASCs have serious restrictions for sampling due to their location, being avoided in malaria clinical trials. This issue has impaired our complete understanding around the immune response triggered by malaria infection in humans.

## Activation of B Cells

Similar to pathogenic infections such as *Trypanosoma cruzi* (the etiologic agent of Chagas disease) or HIV, malaria infection elicits the polyclonal activation of B cells. Among the major factors contributing to this condition are the parasite-specific antigens and cytokines. Consequently, malaria patients present hypergammaglobulinemia, i.e., the increased serologic IgG titers. Furthermore, asymptomatic individuals with high parasitemia usually display broader antibody responses than the asymptomatic individuals with low parasitemia or symptomatic individuals (29). Thus, the malarial parasite load derived from the acute phase of infection seems to drive the ASC response as described for HIV and SIV (30) and Silveira et al., manuscript in preparation. A primary parasite exposure elicits the activation and differentiation of naive B cells into *Plasmodium*-specific MBCs and ASCs. These antigen-specific MBCs, activated through engagement of BCR or Toll-like receptors (31), can also be differentiated into ASCs, enhancing the antibody secretion upon Pf infection (32–35). Alternatively, MBCs induced by *S. mansion* worms may cross-react with Pf antigens and become activated in an malarial-specific manner (36, 37). However, this humoral response does not reach enough concentration in the serum to provide protection. As already mentioned, the individuals from malaria-endemic areas develop an antibody-derived natural immunity only after multiple parasite exposures.

Among the several potential parasite antigens that could induce hypergammaglobulinemia under infection, the domain C1DR1a of EMP1 from a cloned strain (FCR3S1.2), a blood-stage antigen, has been identified in Pf (38). It remains elusive whether the same antigen derived from Pf isolates can also trigger hypergammaglobulinaemia. Interestingly, the domain C1DR1a of PfEMP1 preferentially promotes the MBC activation and proliferation (39). Another *Plasmodium* variant antigen (MSP-1) has been described to activate antigen-specific IgM+ MBCs as the earlier responders upon a malaria re-challenge in mice. Both IgM- and IgG-secreting cells can be generated from the differentiation of those IgM+ MBCs (21). It is still obscure whether the domain C1DR1a is capable of eliciting a similar response during malaria infection in humans. Considering that autoimmunity has been commonly detected during malaria infection as a result of hypergammaglobulinemia, molecular mimicking cannot be ruled out for parasite antigens. In fact, the serum samples of systemic lupus erythematosus (SLE) patients displayed the ability to recognize the Pf malarial antigens (40). Cardiolipin, histones, and DNA are among the auto-antigens usually targeted by the anti-*Plasmodium* antibodies (41).

Another important piece of the polyclonal B cell activation puzzle is related to the modifications in the cytokine profile during malaria infection. BAFF is known to support B cell differentiation into ASCs and potentially elicit hypergammaglobulinemia. Increased levels of this cytokine have been found in the plasma of volunteers upon the Pf challenge (42), in acute Pf-infected children (43), in pregnant women (44), and in Pv infection (45). Moreover, the IL-10 has shown increased levels detected upon the Pf or Pv infections (46) and can influence the serological BAFF levels (47).

### B Cell Subsets

Strikingly, human and murine malaria infections strongly alter the composition of B cell subsets. A murine malaria model showed severe reductions in the bone marrow-derived B cell progenitor numbers (48). Alternately, the infection enhances the numbers of atypical hematopoietic stem and progenitor cells (HSPCs) in the murine spleen. Considering their potential to differentiate into B cells and generate the GC B cells, MBCs, and antigen-specific ASCs *in vivo* (49), the HSPCs could repopulate the immune cell repertoire by counteracting the deficiency in B cell progenitors. Besides progenitor B cells, the Pf and Pv infections also affect the frequency of the peripheral B cells. Whereas the kinetics of classical MBCs and ASCs seem paradoxical in infected individuals living in endemic or non-endemic areas, the transitional B cells (TBCs) and atypical MBCs consistently have shown increased numbers in the blood of all individuals (42, 50).

In terms of TBCs, their frequency has been directly correlated to high parasitemias and an impaired immunity to Pf infection (50). Interestingly, the BAFF receptor signaling has been connected to the control of the immature B cell differentiation to TBCs (51). Considering that BAFF levels are significantly higher during the acute phase of Pf and Pv malaria (43, 45), this cytokine could indeed stimulate a stronger TBC proliferation. Regarding the atypical MBCs in malaria, these cells have been mainly characterized as exhausted cells with a decreased capability to differentiate into ASCs and secrete antibodies (24, 25), as for HIV infection (52). However, the monoclonal antibodies cloned out from those cells majorly recognized the Pf-infected RBCs and neutralized Pf parasites. Furthermore, it has been speculated that their contribution for the neutralizing IgG titers would be similar to the classical MBCs (26). Although total atypical MBCs significantly expand upon Pf or Pv malaria infections, parasitic-specific atypical MBCs still present similar frequencies to classical counterparts (26). In terms of longevity, it remains undetermined whether atypical MBCs represent the majority of the malarial-specific MBCs detected years after parasite exposure in primed individuals without reinfection (17). In infected mice, malarial-specific atypical MBCs were recently associated to short-lived responses that were dependent on the presence of the parasite (53). It would be plausible that the atypical MBCs are high-affinity, somatic hypermutated MBCs in human malaria. Indeed, these cells have been described as the first cells to expand during a recall malaria challenge in mice (21) and both cell subsets can differentiate into IgG+ ASCs, strongly related to malaria immunity.

### Follicular T Helper Cells

To clear microbial infections, the immune system simultaneously triggers cellular and humoral responses that converge to the onset of a long-lasting, protective response. Although it correlates with the titers of high-affinity, class-switched antibodies, B cells are not the unique players in this scenario. In fact, the generation of these antibodies relies on assistance from a particular CD4+ T cell subset called the follicular T helper (TFh) cells. More specifically, the germinal center (GC) B cells physically interact with the activated CD4+ T cells in structures of lymphoid organs called follicles. Within the GCs, B-T cell talk leads to B cell activation, which mature through somatic hypermutations in V(D)J Ig genes as well as the Ig isotype switch, and differentiation into ASCs. Importantly, some TFh cytokines have shown to be critical in this process, such as the IL-6 and IL-21 that regulate B cell survival and cell differentiation.

It has been demonstrated that Pv malaria stimulates the expansion of the TFh cells and secretion of the TFh cytokines (54). Interestingly, Pf infection stimulates a less-functional Th1-like Tfh cell subset, whose function does not correlate to ASC differentiation and antibody secretion. Due to the increased secretion of IFNγ and TNFα during the Pf acute malaria, TFh cell precursors increase the T-bet expression and hinder their differentiation into mature TFh cells (55). Moreover, B cells are also affected by that IFNγ secretion, expressing high levels of T-bet and mainly expanding IgG3 class-switched cells that have the phenotype of atypical MBCs (20). Similarly, this issue is also seen in murine malaria models, implicating lower frequencies of the GC B cells and ASCs as well as the decreased antibody titers. Noteworthily, the murine bone marrow reconstituted with T-bet KO CD4^+^ T cells had the TFh cell functionality restored, followed by the elicitation of GC formation and higher antibody titers (27). Alternately, IL-10 signaling restricts the T-bet expression and rescues the GC formation and antibody responses upon malaria infection in mice (56). Hence, the IL-21 has also demonstrated an important role during this process since the IL-21 KO mice showed decreased numbers of splenic GC B cells and ASCs in the bone marrow, lower antibody titers, and, consequently, a failure to control the parasitemia levels upon challenge (57).

Moreover, other factors can disturb the TFh cell differentiation and influence the effectiveness of humoral responses, such as the expression of particular MHC class II molecules by B cells or co-infections. Regarding the MHC class II expression, humanized mice expressing only HLA-DR0401 as the only MHC class II molecules had impaired antibody responses and could not clear parasitemia after a challenge with a strain causing murine malaria. An expanded subset of the regulatory T cells (Tregs) was found to interact with B cells in those mice, rather than the TFh cells. However, the Treg depletion or HLA-DR0401 co-expression with murine MHC class II molecules boosted the antibody titers and, consequently, the parasitemia dropped to undetectable levels in these mice (58). The expression of T-bet and the influence of Th1 cytokines, such as IFNγ, have been associated with that Treg expansion which impairs the Tfh cell differentiation and survival as well as wanes the secretion of malaria-specific antibodies (59, 60). In terms of co-infections, an acute gammaherpesvirus (MHV68) infection decreased the resistance against a non-lethal malaria in mice. This co-infection diminished the frequencies of the Tfh cells, GC B cells, and ASCs, suppressing the humoral response to malaria (61).

### Antibody-dependent Protective Immunity

The malarial-specific antibody responses derived from Pf exposure are usually transient since their titers decrease by the next parasitic transmission season. After their contraction, the antibody levels are still maintained in a higher magnitude than the respective titers detected in the previous parasite exposure (62). For the Pv infection, it follows an opposite pattern (17, 54). However, once secreted at a certain level in the serum, the antibodies can provide protection against human malaria. Serology data against blood stage antigens have determined an inverse correlation between the antibody titers specific for Pf MSP-2-, MSP-3-, and AMA-1 and Pf morbidity in the infected individuals. Thus, an increased breadth of antibody specificity would be associated with a lower chance to experience a clinical episode or be admitted to hospital with severe Pf malaria (63).

To provide antibody-dependent protection, the immune system launches different mechanisms of action upon malaria infection. Considering that the blood stage of infection breaks humoral immunity against the liver stage antigens in murine malaria models (16), malaria-specific antibodies would preferentially opsonize the merozoites. Subsequently, they could trigger effector functions, such as inhibition of cell invasion, phagocytosis, activation of respiratory burst, or complement-derived parasite death [reviewed by (64)]. In the context of the inhibition of cell invasion, it has been challenging to study the breadth of antibody responses which could block sporozoite or merozoite invasion into the hepatocytes or erythrocytes (Pf) / reticulocytes (Pv), respectively. To assess the antigens linked to protective humoral responses against Pf malaria, the high-throughput technologies have been utilized. Among the identified antigens were liver and blood-stage antigens (65). Regarding the reactivity against the *Plasmodium* liver stage antigens, most of such response is driven to the circumsporozoite protein (CSP). The most immunogenic region of Pf CSP for antibodies consists of the central repeat-region. Recently, a Pf CSP-specific mAb (CIS43) displayed a high capacity of parasite neutralization, with its binding region identified in the junction between the N-terminal and central repeat regions of CSP (66). Another anti-Pf CSP repeat-region mAb (2A10) has been shown to elicit protection in mice challenged with chimeric Pb-Pf sporozoites. After being cloned into a adeno-associated virus vector, 2A10 was expressed for long-term and reduced parasite burden, providing protection in mice either by sporozoite injection or mosquito bites (67). The N-terminal region flanking those Pf CSP repeat-regions also possess a linear protective B cell epitope recognized by the mAb 5D5. The antibody-binding inhibits the CSP proteolytic cleavage, neutralizing the hepatocyte invasion. Whenever administrated in combination with mAb 2A10, these mAbs enhanced the sporozoite neutralization *in vivo* (68). Contrarily, the antibody reactivity had multiple targets against the blood stage antigens. Anti-EBA-175 mAbs (R217 and R218) have been described as inhibitory for Pf invasion in RBCs. Whereas R217, the more inhibitory mAb, engages fundamental antigen residues for RBC binding, R218 interacts with F1 region residues, irrelevant for RBC binding (69). The subdomains I and II of Duffy binding protein (DBP) have also been targeted by the neutralizing antibodies detected in high concentration in the serum of individuals from Pv malaria-endemic areas. Mutations in those antibody sequences accumulate over parasitic exposures, enhancing their breadth and potency (70). Furthermore, although barely recognized during infection even in residents of Pf malaria-endemic areas (71), the anti-RH5 antibodies have demonstrated a great capacity of inhibiting invasion of the Pf merozoites in erythrocytes (72–76). Due to a subcellular location, the RH5 antigen has been detected around the moving junction that is assembled just before the erythrocyte invasion. Thus, RH5 would be accessible to antibodies only during the short contact between Pf merozoites and erythrocytes. Crystallography data showed that anti-RH5 bNabs bind at or close to the basigin-binding site, blocking the interaction between RH5 and basigin (77) that is critical for Pf merozoite invasion. However, this protein interaction does not seem to be the unique spot for the anti-RH5 mAb neutralizing activity. Considering that distinct RH5-derived B cell epitopes have been described with those bNAbs, it suggests that the RH5 sequences may suffer some immunological pressure (78). Other Pf antigens have been described as stimulators of malaria bNAbs, such as EXP1, MSP-3, GLURP, RAMA, SEA, and EBA-181. Those antigens were discovered after an investigation of serum samples from the cured Pf malaria patients and individuals with subsequent recrudescent infection. The cured patient samples had higher antibody titers against all those antigens and consequently, a higher capacity to inhibit the erythrocytes invasion by Pf merozoites (79). Recently, a mechanism based on interchromosomal DNA transposition was described as the contributor to the antibody diversity in the context of Pf malaria infection. A DNA insertion from a sequence of a collagen-binding inhibitory receptor (LAIR1) into V(D)J Ig genes was described to generate broad reactive antibodies against the Pf-infected erythrocytes (80). Although these LAIR1-containing antibodies were found in 5-10% of residents of Pf malaria-endemic areas and recognized distinct members of the RIFIN family, they did not confer protection against the disease (80, 81).

Similar to the importance of immunoglobulin variable regions for effective humoral responses, Fc regions also have a fundamental role in mediating protection against infectious diseases. Receptors for immunoglobulin Fc regions have been described to be involved in cellular processes such as phagocytosis, antibody-dependent cellular cytotoxicity, and inflammation, among others (82). In the context of Pf malaria infection, the inoculations of human anti-MSP1_19_ IgG protected transgenic mice for human Fc gamma receptor after challenge with chimeric Pb-Pf sporozoites. Contrarily, protection was not obtained when the same mAb was tested in non-transgenic mice, suggesting that the antibody interaction with MSP1_19_ is not sufficient, while the presence of the Fc region is critical for parasite clearance (83). Few studies about human single nucleotide polymorphisms (SNPs) of the Fc gamma receptor have already validated the relevance of this opsonizing antibody-dependent phagocytosis for Pf malaria immunity [reviewed by (64)]. Noteworthily, the merozoite opsonisation has been associated with several conserved antigens and induces immunity against multiple Pf parasite strains (84).

Among the cells that can phagocyte and eliminate an opsonised Pf merozoite are neutrophils through the activation of respiratory burst [reviewed by (64)]. A SNP study has associated the increased levels of nitric oxide (NO) to Pf malaria protection (85). Furthermore, ROS levels have been correlated to natural acquired Pf malaria immunity (86). Once secreted to the extracellular medium, NO and ROS can both dampen the growth of Pf parasites *in vitro* (64, 87).

In terms of antibody-dependent complement activation, complement C1q proteins have shown to be deposited on the merozoite surface after the antibody-antigen interaction, allowing the further formation of the membrane attack complex (MAC) for parasite destruction. MSP-1 and MSP-2 have been identified as the main Pf merozoite targets involved in this protective mechanism. The higher is the C1q deposition, the higher is the protection. Interestingly, older children from Pf endemic areas presented higher C1q deposition than the younger children (88).

## Concluding Remarks

**Figure 1:**
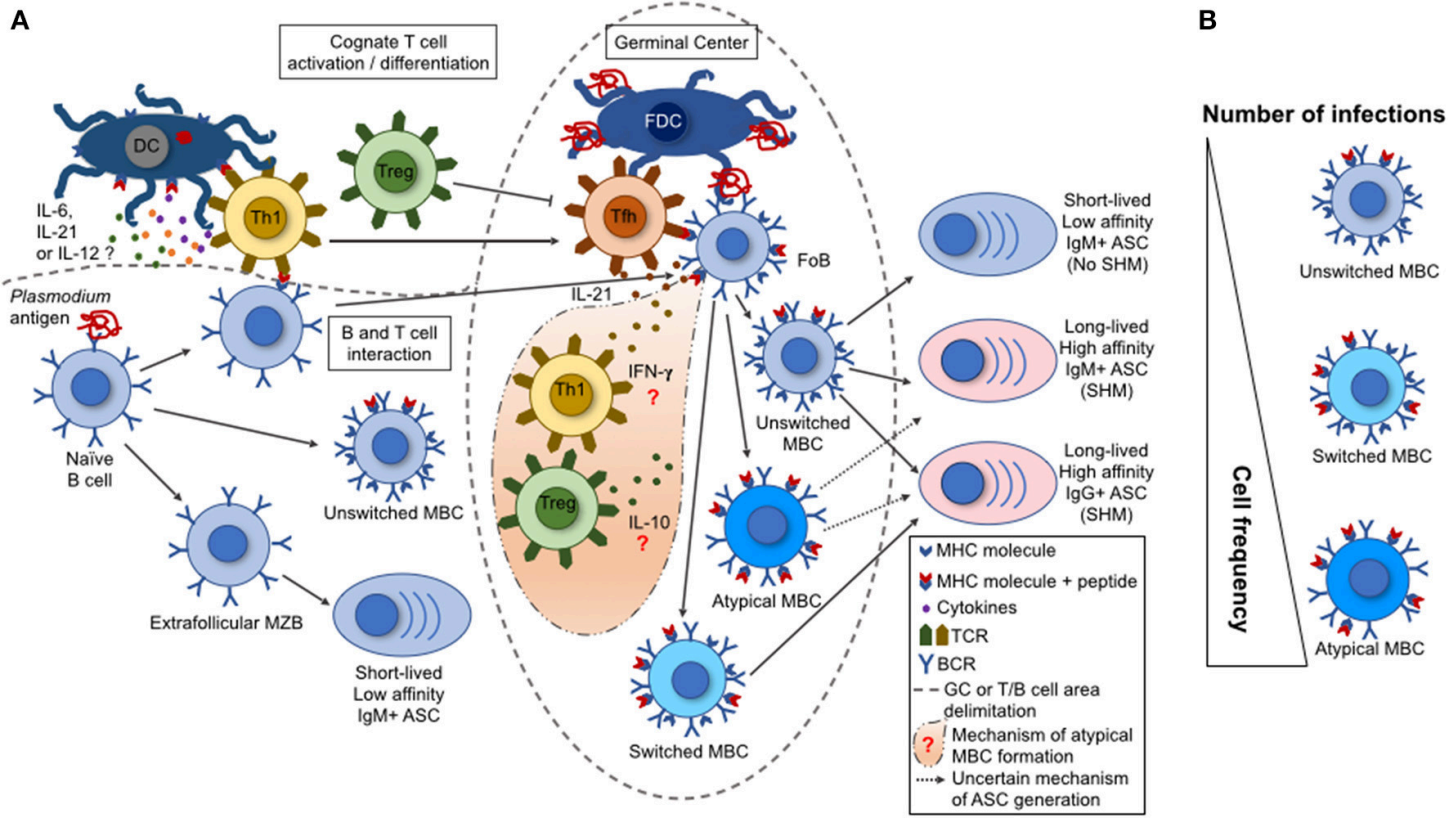
B cell response triggered by malaria infection. **(A)** During a malaria infection, the naive B cells are activated by a *Plasmodium* antigen through the interaction with B cell receptors (BCR), leading to their differentiation into marginal zone B cells (MZB), follicular B cells (FoB), or unswitched memory B cell (MBCs). The switched and atypical MBCs are derived from the activation of FoBs within the germinal centers (GCs). Either the MZBs, or the unswitched, switched, or atypical MBCs can differentiate into antibody-secreting cells (ASCs). These ASCs range from short-lived, low-affinity, IgM+ to long-lived, high-affinity, IgM+ or IgG+. This variation depends on the type of interaction between a particular B cell with a T cell subset. The activated Th1 T cells migrate to the GCs, becoming follicular T helper cells (TFh) that help the GC reactions (acquisition of somatic hypermutations in V(D)J Ig genes and class switch by activated FoBs). Contrarily, the regulatory T cells (Tregs) have the potential to inhibit TFh cell differentiation and GC reactions. **(B)** A single parasite infection can induce the differentiation of multiple *Plasmodium*-specific B cell clones. However, the repeated parasite exposures shift the MBC frequencies with an increase for an atypical MBC over the unswitched or switched MBCs. This shift in cell frequency may interfere on the function of the secreted antibodies and, consequently, on the development of protective immunity.

After decades of suffering with malaria morbidity and mortality in several tropical areas, the at risk-populations are long overdue for effective strategies to contain this epidemic. The development of *Plasmodium*-specific vaccines already tested in clinical trials has not presented a considerable degree of protection yet. Hence, it has been strongly demonstrated that *Plasmodium* parasites have an enhanced resistance against the anti-malarial drugs on a daily basis. Thus, other alternatives of preventative or therapeutic treatments for malaria should be considered. Following all the characteristics presented in this manuscript about malaria-derived B cell immunity (Figure 1), those formulations must prioritize the best conditions for optimal B cell activation and development of the GC, TFh, and ASC responses. In terms of antibodies which are the final and effector products of the potential formulations, there are several reports of neutralizing antibodies identified for human malaria. However, their mechanisms of action have not been elucidated yet. Overall, it has been widely seen that antibody-based prophylaxis or therapeutical approaches possess a great efficacy against multiple pathogens. Therefore, strategies that properly stimulate malarial B cell responses may be beneficial not only in inhibiting infection, but also in reducing the morbidity and mortality numbers and disease transmission.

## Author Contributions

ELVS and MRD wrote the draft of this manuscript. ISS reviewed the manuscript and contributed significantly to the final draft. All authors read and approved the final manuscript.

### Conflict of interest statement

The authors declare that the research was conducted in the absence of any commercial or financial relationships that could be construed as a potential conflict of interest.

## References

[1] World Health Organization World Malaria Report 2016. (2016). Available from: http://www.who.int/malaria/publications/world-malaria-report-2016/report/en/ (Accessed February 14, 2017).

[2] World Health Organization Management of Severe Malaria: A Practical Handbook. Geneva (2012). p. 1–83.

[3] Lacerda MV Mourão MP Alexandre MA Siqueira AM Magalhães BM Martinez-Espinosa FE. Understanding the clinical spectrum of complicated *Plasmodium vivax* malaria: a systematic review on the contributions of the Brazilian literature. Malar J. (2012) 11:12. 10.1186/1475-2875-11-12 22230294 PMC3268102

[4] Murray CJ Rosenfeld LC Lim SS Andrews KG Foreman KJ Haring D. Global malaria mortality between 1980 and 2010: a systematic analysis. Lancet (2012) 379:413–31. 10.1016/S0140-6736(12)60034-8 22305225

[5] Ménard R Tavares J Cockburn I Markus M Zavala F Amino R. Looking under the skin: the first steps in malarial infection and immunity. Nat Rev Microbiol. (2013) 11:701–12. 10.1038/nrmicro3111 24037451

[6] Absalon S Robbins JA Dvorin JD. An essential malaria protein defines the architecture of blood-stage and transmission-stage parasites. Nat Commun. (2016) 7:11449. 10.1038/ncomms11449 27121004 PMC4853479

[7] Obaldia N Meibalan E Sa JM Ma S Clark MA Mejia P. Bone marrow is a major parasite reservoir in *Plasmodium vivax* infection. MBio (2018) 9:e00625-18. 10.1128/mBio.00625-18 29739900 PMC5941073

[8] Joice R Nilsson SK Montgomery J Dankwa S Egan E Morahan B. Plasmodium falciparum transmission stages accumulate in the human bone marrow. Sci Transl Med. (2014) 6:244re5. 10.1126/scitranslmed.3008882 25009232 PMC4175394

[9] Cohen S McGregor IA Carrington S. Gamma-globulin and acquired immunity to human malaria. Nature (1961) 192:733–7. 10.1038/192733a0 13880318

[10] Ferreira MU da Silva Nunes M Wunderlich G. Antigenic diversity and immune evasion by malaria parasites. Clin Diagn Lab Immunol. (2004) 11:987–95. 10.1128/CDLI.11.6.987-995.2004 15539495 PMC524792

[11] Neafsey DE Galinsky K Jiang RH Young L Sykes SM Saif S. The malaria parasite *Plasmodium vivax* exhibits greater genetic diversity than *Plasmodium falciparum*. Nat Genet. (2012) 44:1046–50. 10.1038/ng.2373 22863733 PMC3432710

[12] Weiss GE Traore B Kayentao K Ongoiba A Doumbo S Doumtabe D. The Plasmodium falciparum-specific human memory B cell compartment expands gradually with repeated malaria infections. PLoS Pathog. (2010) 6:e1000912. 10.1371/journal.ppat.1000912 20502681 PMC2873912

[13] Nogaro SI Hafalla JC Walther B Remarque EJ Tetteh KK Conway DJ The breadth, but not the magnitude, of circulating memory B cell responses to *P*. falciparum increases with age/exposure in an area of low transmission. PLoS ONE (2011) 6:e25582 10.1371/journal.pone.0025582 21991321 PMC3186790

[14] Wrammert J Smith K Miller J Langley WA Kokko K Larsen C. Rapid cloning of high-affinity human monoclonal antibodies against influenza virus. Nature (2008) 453:667–71. 10.1038/nature06890 18449194 PMC2515609

[15] Le Roch KG Johnson JR Florens L Zhou Y Santrosyan A Grainger M Global analysis of transcript and protein levels across the *Plasmodium falciparum* life cycle. Genome Res. (2004) 14:2308–18. 10.1101/gr.2523904 15520293 PMC525690

[16] Keitany GJ Kim KS Krishnamurty AT Hondowicz BD Hahn WO Dambrauskas N. Blood stage malaria disrupts humoral immunity to the pre-erythrocytic stage circumsporozoite protein. Cell Rep. (2016) 17:3193–205. 10.1016/j.celrep.2016.11.060 28009289 PMC5476299

[17] Wipasa J Suphavilai C Okell LC Cook J Corran PH Thaikla K. Long-lived antibody and B Cell memory responses to the human malaria parasites, *Plasmodium falciparum* and *Plasmodium vivax*. PLoS Pathog. (2010) 6:e1000770. 10.1371/journal.ppat.1000770 20174609 PMC2824751

[18] Ubillos I Campo JJ Requena P Ome-Kaius M Hanieh S Rose H. Chronic exposure to malaria is associated with inhibitory and activation markers on atypical memory B cells and marginal zone-like B cells. Front Immunol. (2017) 8:966. 10.3389/fimmu.2017.00966 28878766 PMC5573441

[19] Stephens R Ndungu FM Langhorne J. Germinal centre and marginal zone B cells expand quickly in a second *Plasmodium chabaudi* malaria infection producing mature plasma cells. Parasite Immunol. (2009) 31:20–31. 10.1111/j.1365-3024.2008.01066.x 19121080 PMC2680269

[20] Obeng-Adjei N Portugal S Holla P Li S Sohn H Ambegaonkar A. Malaria-induced interferon-γ drives the expansion of Tbethi atypical memory B cells. PLoS Pathog. (2017) 13:e1006576. 10.1371/journal.ppat.1006576 28953967 PMC5633206

[21] Krishnamurty AT Thouvenel CD Portugal S Keitany GJ Kim KS Holder A. Somatically hypermutated plasmodium-specific IgM(+) memory B cells are rapid, plastic, early responders upon malaria rechallenge. Immunity (2016) 45:402–14. 10.1016/j.immuni.2016.06.014 27473412 PMC5118370

[22] Harms Pritchard G Pepper M Memory B cell heterogeneity: remembrance of things past. J Leukoc Biol. (2018) 103:269–74. 10.1002/JLB.4MR0517-215R 29345369 PMC6200418

[23] Radbruch A Muehlinghaus G Luger EO Inamine A Smith KG Dörner T. Competence and competition: the challenge of becoming a long-lived plasma cell. Nat Rev Immunol. (2006) 6:741–50. 10.1038/nri1886 16977339

[24] Weiss GE Crompton PD Li S Walsh LA Moir S Traore B. Atypical memory B cells are greatly expanded in individuals living in a malaria-endemic area. J Immunol. (2009) 183:2176–82. 10.4049/jimmunol.0901297 19592645 PMC2713793

[25] Portugal S Tipton CM Sohn H Kone Y Wang J Li S. Malaria-associated atypical memory B cells exhibit markedly reduced B cell receptor signaling and effector function. Elife (2015) 4:e07218. 10.7554/eLife.07218 25955968 PMC4444601

[26] Muellenbeck MF Ueberheide B Amulic B Epp A Fenyo D Busse CE. Atypical and classical memory B cells produce *Plasmodium falciparum* neutralizing antibodies. J Exp Med. (2013) 210:389–99. 10.1084/jem.20121970 23319701 PMC3570107

[27] Ryg-Cornejo V Ioannidis LJ Ly A Chiu CY Tellier J Hill DL. Severe malaria infections impair germinal center responses by inhibiting T follicular helper cell differentiation. Cell Rep. (2016) 14:68–81. 10.1016/j.celrep.2015.12.006 26725120

[28] Xue J Zhan B Guo J He N Qiang HQ Hotez P. Acquired hookworm immunity in the golden hamster (*Mesocricetus auratus*) elicited by living *Necator americanus* third-stage infective larvae. Exp Parasitol. (2012) 130:6–12. 10.1016/j.exppara.2011.10.007 22024448

[29] Finney OC Danziger SA Molina DM Vignali M Takagi A Ji M. Predicting antidisease immunity using proteome arrays and sera from children naturally exposed to malaria. Mol Cell Proteomics (2014) 13:2646–60. 10.1074/mcp.M113.036632 25023128 PMC4188993

[30] Cepok S von Geldern G Nolting T Grummel V Srivastava R Zhou D. Viral load determines the B-cell response in the cerebrospinal fluid during human immunodeficiency virus infection. Ann Neurol. (2007) 62:458–67. 10.1002/ana.21195 17703460

[31] Crompton PD Mircetic M Weiss G Baughman A Huang CY Topham DJ. The TLR9 ligand CpG promotes the acquisition of *Plasmodium falciparum*-specific memory B cells in malaria-naive individuals. J Immunol. (2009) 182:3318–26. 10.4049/jimmunol.0803596 19234231 PMC2910392

[32] Elias SC Choudhary P de Cassan SC Biswas S Collins KA Halstead FD. Analysis of human B-cell responses following ChAd63-MVA MSP1 and AMA1 immunization and controlled malaria infection. Immunology (2014) 141:628–44. 10.1111/imm.12226 24303947 PMC3956436

[33] Turner L Wang CW Lavstsen T Mwakalinga SB Sauerwein RW Hermsen CC. Antibodies against PfEMP1, RIFIN, MSP3 and GLURP are acquired during controlled Plasmodium falciparum malaria infections in naïve volunteers. PLoS ONE. (2011) 6:e29025. 10.1371/journal.pone.0029025 22174947 PMC3236238

[34] Walker DM Oghumu S Gupta G McGwire BS Drew ME Satoskar AR. Mechanisms of cellular invasion by intracellular parasites. Cell Mol Life Sci. (2014) 71:1245–63. 10.1007/s00018-013-1491-1 24221133 PMC4107162

[35] Walker KM Okitsu S Porter DW Duncan C Amacker M Pluschke G. Antibody and T-cell responses associated with experimental human malaria infection or vaccination show limited relationships. Immunology (2015) 145:71–81. 10.1111/imm.12428 25471322 PMC4405325

[36] Naus CW Jones FM Satti MZ Joseph S Riley EM Kimani G. Serological responses among individuals in areas where both schistosomiasis and malaria are endemic: cross-reactivity between *Schistosoma mansoni* and *Plasmodium falciparum*. J Infect Dis. (2003) 187:1272–82. 10.1086/368361 12696007

[37] Pierrot C Wilson S Lallet H Lafitte S Jones FM Daher W. Identification of a novel antigen of *Schistosoma mansoni* shared with *Plasmodium falciparum* and evaluation of different cross-reactive antibody subclasses induced by human schistosomiasis and malaria. Infect Immun. (2006) 74:3347–54. 10.1128/IAI.01724-05 16714563 PMC1479256

[38] Donati D Zhang LP Chêne A Chen Q Flick K Nyström M. Identification of a polyclonal B-cell activator in *Plasmodium falciparum*. Infect Immun. (2004) 72:5412–8. 10.1128/IAI.72.9.5412-5418.2004 15322039 PMC517431

[39] Donati D Mok B Chêne A Xu H Thangarajh M Glas R. Increased B cell survival and preferential activation of the memory compartment by a malaria polyclonal B cell activator. J Immunol. (2006) 177:3035–44. 10.4049/jimmunol.177.5.3035 16920940

[40] Zanini GM De Moura Carvalho LJ Brahimi K De Souza-Passos LF Guimarães SJ Da Silva Machado E. Sera of patients with systemic lupus erythematosus react with plasmodial antigens and can inhibit the *in vitro* growth of *Plasmodium falciparum*. Autoimmunity (2009) 42:545–52. 10.1080/08916930903039810 19657771

[41] Brahimi K Martins YC Zanini GM Ferreira-da-Cruz MeF Daniel-Ribeiro CT. Monoclonal auto-antibodies and sera of autoimmune patients react with *Plasmodium falciparum* and inhibit its *in vitro* growth. Mem Inst Oswaldo Cruz. (2011) 106(Suppl. 1):44–51. 10.1590/S0074-02762011000900006 21881756

[42] Scholzen A Teirlinck AC Bijker EM Roestenberg M Hermsen CC Hoffman SL. BAFF and BAFF receptor levels correlate with B cell subset activation and redistribution in controlled human malaria infection. J Immunol. (2014) 192:3719–29. 10.4049/jimmunol.1302960 24646735 PMC4028688

[43] Nduati E Gwela A Karanja H Mugyenyi C Langhorne J Marsh K. The plasma concentration of the B cell activating factor is increased in children with acute malaria. J Infect Dis. (2011) 204:962–70. 10.1093/infdis/jir438 21849293 PMC3156925

[44] Muehlenbachs A Fried M Lachowitzer J Mutabingwa TK Duffy PE. Genome-wide expression analysis of placental malaria reveals features of lymphoid neogenesis during chronic infection. J Immunol. (2007) 179:557–65. 10.4049/jimmunol.179.1.557 17579077

[45] Patgaonkar M Herbert F Powale K Gandhe P Gogtay N Thatte U. Vivax infection alters peripheral B-cell profile and induces persistent serum IgM. Parasite Immunol. (2018) 40:e12580. 10.1111/pim.12580 30102786

[46] Rodrigues-da-Silva RN Lima-Junior JaC Fonseca BeP Antas PR Baldez A Storer FL. Alterations in cytokines and haematological parameters during the acute and convalescent phases of *Plasmodium falciparum* and *Plasmodium vivax* infections. Mem Inst Oswaldo Cruz. (2014) 109:154–62. 10.1590/0074-0276140275 24676654 PMC4015248

[47] Craxton A Magaletti D Ryan EJ Clark EA. Macrophage- and dendritic cell–dependent regulation of human B-cell proliferation requires the TNF family ligand BAFF. Blood (2003) 101:4464–71. 10.1182/blood-2002-10-3123 12531790

[48] Bockstal V Geurts N Magez S. Acute Disruption of Bone Marrow B Lymphopoiesis and apoptosis of transitional and marginal zone B cells in the spleen following a blood-stage *Plasmodium chabaudi* infection in mice. J Parasitol Res. (2011) 2011:534697. 10.1155/2011/534697 21687602 PMC3112522

[49] Ghosh D Wikenheiser DJ Kennedy B McGovern KE Stuart JD Wilson EH. An atypical splenic B cell progenitor population supports antibody production during plasmodium infection in mice. J Immunol. (2016) 197:1788–800. 10.4049/jimmunol.1502199 27448588 PMC4992648

[50] Sullivan RT Ssewanyana I Wamala S Nankya F Jagannathan P Tappero JW B cell sub-types following acute malaria and associations with clinical immunity. Malar J. (2016) 15:139 10.1186/s12936-016-1190-0 26939776 PMC4778296

[51] Rowland SL Leahy KF Halverson R Torres RM Pelanda R. BAFF receptor signaling aids the differentiation of immature B cells into transitional B cells following tonic BCR signaling. J Immunol. (2010) 185:4570–81. 10.4049/jimmunol.1001708 20861359 PMC2950883

[52] Moir S Ho J Malaspina A Wang W DiPoto AC O'Shea MA. Evidence for HIV-associated B cell exhaustion in a dysfunctional memory B cell compartment in HIV-infected viremic individuals. J Exp Med. (2008) 205:1797–805. 10.1084/jem.20072683 18625747 PMC2525604

[53] Pérez-Mazliah D Gardner PJ Schweighoffer E McLaughlin S Hosking C Tumwine I. Plasmodium-specific atypical memory B cells are short-lived activated B cells. Elife (2018) 7:e39800. 10.7554/eLife.39800 30387712 PMC6242553

[54] Figueiredo MM Costa PAC Diniz SQ Henriques PM Kano FS Tada MS. T follicular helper cells regulate the activation of B lymphocytes and antibody production during *Plasmodium vivax* infection. PLoS Pathog. (2017) 13:e1006484. 10.1371/journal.ppat.1006484 28700710 PMC5519210

[55] Obeng-Adjei N Portugal S Tran TM Yazew TB Skinner J Li S. Circulating Th1-cell-type Tfh cells that exhibit impaired B cell help are preferentially activated during acute malaria in children. Cell Rep. (2015) 13:425–39. 10.1016/j.celrep.2015.09.004 26440897 PMC4607674

[56] Guthmiller JJ Graham AC Zander RA Pope RL Butler NS. Cutting Edge: IL-10 is essential for the generation of germinal center B cell responses and anti-plasmodium humoral immunity. J Immunol. (2017) 198:617–22. 10.4049/jimmunol.1601762 27940658 PMC5225073

[57] Pérez-Mazliah D Ng DH Freitas do Rosário AP McLaughlin S Mastelic-Gavillet B Sodenkamp J. Disruption of IL-21 signaling affects T cell-B cell interactions and abrogates protective humoral immunity to malaria. PLoS Pathog. (2015) 11:e1004715. 10.1371/journal.ppat.1004715 25763578 PMC4370355

[58] Wijayalath W Danner R Kleschenko Y Majji S Villasante EF Richie TL. HLA class II (DR0401) molecules induce Foxp3+ regulatory T cell suppression of B cells in *Plasmodium yoelii* strain 17XNL malaria. Infect Immun. (2014) 82:286–97. 10.1128/IAI.00272-13 24166949 PMC3911852

[59] Zander RA Obeng-Adjei N Guthmiller JJ Kulu DI Li J Ongoiba A. PD-1 co-inhibitory and OX40 co-stimulatory crosstalk regulates helper T cell differentiation and anti-plasmodium humoral immunity. Cell Host Microbe (2015) 17:628–41. 10.1016/j.chom.2015.03.007 25891357 PMC4433434

[60] Zander RA Guthmiller JJ Graham AC Pope RL Burke BE Carr DJ. Type I interferons induce T regulatory 1 responses and restrict humoral immunity during experimental malaria. PLoS Pathog. (2016) 12:e1005945. 10.1371/journal.ppat.1005945 27732671 PMC5061386

[61] Matar CG Anthony NR O'Flaherty BM Jacobs NT Priyamvada L Engwerda CR. Gammaherpesvirus co-infection with malaria suppresses anti-parasitic humoral immunity. PLoS Pathog. (2015) 11:e1004858. 10.1371/journal.ppat.1004858 25996913 PMC4440701

[62] Hviid L Barfod L Fowkes FJ. Trying to remember: immunological B cell memory to malaria. Trends Parasitol. (2015) 31:89–94. 10.1016/j.pt.2014.12.009 25596801

[63] Osier FH Fegan G Polley SD Murungi L Verra F Tetteh KK. Breadth and magnitude of antibody responses to multiple *Plasmodium falciparum* merozoite antigens are associated with protection from clinical malaria. Infect Immun. (2008) 76:2240–8. 10.1128/IAI.01585-07 18316390 PMC2346713

[64] Hill DL Schofield L Wilson DW. IgG opsonization of merozoites: multiple immune mechanisms for malaria vaccine development. Int J Parasitol. (2017) 47:585–95. 10.1016/j.ijpara.2017.05.004 28668325

[65] Dent AE Nakajima R Liang L Baum E Moormann AM Sumba PO. *Plasmodium falciparum* protein microarray antibody profiles correlate with protection from symptomatic malaria in Kenya. J Infect Dis. (2015) 212:1429–38. 10.1093/infdis/jiv224 25883384 PMC4601912

[66] Kisalu NK Idris AH Weidle C Flores-Garcia Y Flynn BJ Sack BK. A human monoclonal antibody prevents malaria infection by targeting a new site of vulnerability on the parasite. Nat Med. (2018) 24:408–16. 10.1038/nm.4512 29554083 PMC5893371

[67] Deal C Balazs AB Espinosa DA Zavala F Baltimore D Ketner G. Vectored antibody gene delivery protects against *Plasmodium falciparum* sporozoite challenge in mice. Proc Natl Acad Sci USA. (2014) 111:12528–32. 10.1073/pnas.1407362111 25114213 PMC4151717

[68] Espinosa DA Gutierrez GM Rojas-López M Noe AR Shi L Tse SW. Proteolytic cleavage of the *Plasmodium falciparum* circumsporozoite protein is a target of protective antibodies. J Infect Dis. (2015) 212:1111–9. 10.1093/infdis/jiv154 25762791 PMC4559189

[69] Chen E Paing MM Salinas N Sim BK Tolia NH. Structural and functional basis for inhibition of erythrocyte invasion by antibodies that target *Plasmodium falciparum* EBA-175. PLoS Pathog. (2013) 9:e1003390. 10.1371/journal.ppat.1003390 23717209 PMC3662668

[70] Chootong P Ntumngia FB VanBuskirk KM Xainli J Cole-Tobian JL Campbell CO. Mapping epitopes of the *Plasmodium vivax* Duffy binding protein with naturally acquired inhibitory antibodies. Infect Immun. (2010) 78:1089–95. 10.1128/IAI.01036-09 20008533 PMC2825952

[71] Partey FD Castberg FC Sarbah EW Silk SE Awandare GA Draper SJ Kinetics of antibody responses to PfRH5-complex antigens in Ghanaian children with *Plasmodium falciparum* malaria. PLoS ONE (2018) 13:e0198371 10.1371/journal.pone.0198371 29883485 PMC5993283

[72] Douglas AD Williams AR Illingworth JJ Kamuyu G Biswas S Goodman AL. The blood-stage malaria antigen PfRH5 is susceptible to vaccine-inducible cross-strain neutralizing antibody. Nat Commun. (2011) 2:601. 10.1038/ncomms1615 22186897 PMC3504505

[73] Douglas AD Baldeviano GC Lucas CM Lugo-Roman LA Crosnier C Bartholdson SJ. A PfRH5-based vaccine is efficacious against heterologous strain blood-stage Plasmodium falciparum infection in aotus monkeys. Cell Host Microbe (2015) 17:130–9. 10.1016/j.chom.2014.11.017 25590760 PMC4297294

[74] Weaver R Reiling L Feng G Drew DR Mueller I Siba PM. The association between naturally acquired IgG subclass specific antibodies to the PfRH5 invasion complex and protection from *Plasmodium falciparum malaria*. Sci Rep. (2016) 6:33094. 10.1038/srep33094 27604417 PMC5015043

[75] Kapulu MC Da DF Miura K Li Y Blagborough AM Churcher TS. Comparative assessment of transmission-blocking vaccine candidates against *Plasmodium falciparum*. Sci Rep. (2015) 5:11193. 10.1038/srep11193 26063320 PMC4463016

[76] Payne RO Silk SE Elias SC Miura K Diouf A Galaway F. Human vaccination against RH5 induces neutralizing antimalarial antibodies that inhibit RH5 invasion complex interactions. JCI Insight. (2017) 2:96381. 10.1172/jci.insight.96381 29093263 PMC5752323

[77] Wright KE Hjerrild KA Bartlett J Douglas AD Jin J Brown RE. Structure of malaria invasion protein RH5 with erythrocyte basigin and blocking antibodies. Nature (2014) 515:427–30. 10.1038/nature13715 25132548 PMC4240730

[78] Douglas AD Williams AR Knuepfer E Illingworth JJ Furze JM Crosnier C. Neutralization of *Plasmodium falciparum* merozoites by antibodies against PfRH5. J Immunol. (2014) 192:245–58. 10.4049/jimmunol.1302045 24293631 PMC3872115

[79] Goh YS Peng K Chia WN Siau A Chotivanich K Gruner AC. Neutralizing antibodies against *Plasmodium falciparum* associated with successful cure after drug therapy. PLoS ONE (2016) 11:e0159347. 10.1371/journal.pone.0159347 27427762 PMC4948787

[80] Tan J Pieper K Piccoli L Abdi A Perez MF Geiger R. A LAIR1 insertion generates broadly reactive antibodies against malaria variant antigens. Nature (2016) 529:105–9. 10.1038/nature16450 26700814 PMC4869849

[81] Pieper K Tan J Piccoli L Foglierini M Barbieri S Chen Y. Public antibodies to malaria antigens generated by two LAIR1 insertion modalities. Nature (2017) 548:597–601. 10.1038/nature23670 28847005 PMC5635981

[82] Pleass RJ Holder AA. Opinion: antibody-based therapies for malaria. Nat Rev Microbiol. (2005) 3:893–9. 10.1038/nrmicro1267 16261172

[83] McIntosh RS Shi J Jennings RM Chappel JC de Koning-Ward TF Smith T. The importance of human FcgammaRI in mediating protection to malaria. PLoS Pathog. (2007) 3:e72. 10.1371/journal.ppat.0030072 17511516 PMC1868954

[84] Hill DL Wilson DW Sampaio NG Eriksson EM Ryg-Cornejo V Harrison GLA. Merozoite antigens of *Plasmodium falciparum* elicit strain-transcending opsonizing immunity. Infect Immun. (2016) 84:2175–84. 10.1128/IAI.00145-16 27185785 PMC4962632

[85] Kun JF Mordmüller B Perkins DJ May J Mercereau-Puijalon O Alpers M. Nitric oxide synthase 2(Lambaréné) (G-954C), increased nitric oxide production, and protection against malaria. J Infect Dis. (2001) 184:330–6. 10.1086/322037 11443559

[86] Joos C Marrama L Polson HE Corre S Diatta AM Diouf B. Clinical protection from falciparum malaria correlates with neutrophil respiratory bursts induced by merozoites opsonized with human serum antibodies. PLoS ONE (2010) 5:e9871. 10.1371/journal.pone.0009871 20360847 PMC2845614

[87] Hodgson SH Llewellyn D Silk SE Milne KH Elias SC Miura K. Changes in serological immunology measures in UK and Kenyan adults post-controlled human malaria infection. Front Microbiol. (2016) 7:1604. 10.3389/fmicb.2016.01604 27790201 PMC5061779

[88] Boyle MJ Reiling L Feng G Langer C Osier FH Aspeling-Jones H. Human antibodies fix complement to inhibit *Plasmodium falciparum* invasion of erythrocytes and are associated with protection against malaria. Immunity (2015) 42:580–90. 10.1016/j.immuni.2015.02.012 25786180 PMC4372259

